# Assessing phototaxis behavior in non-resistant and insecticide-resistant populations of *Hyalella azteca*

**DOI:** 10.1007/s10646-026-03084-x

**Published:** 2026-04-24

**Authors:** Nick Hettel, Cristina G.B. La, Helen C. Poynton, Giovanni S. Molinari, Kara E. Huff Hartz, Michael J. Lydy

**Affiliations:** 1 Center for Fisheries, Aquaculture and Aquatic Sciences, Department of Zoology, Southern Illinois University, Carbondale, IL 62901 USA; 2 School of Biological Sciences, Southern Illinois University, Carbondale, IL 62901 USA; 3 School for the Environment, University of Massachusetts Boston, Boston, MA 02125 USA

**Keywords:** *Hyalella azteca*, Behavior, Fitness, Pyrethroids, Insecticide-resistance, Phototaxis

## Abstract

Pyrethroid insecticides are ubiquitous in waterways throughout the United States. Consequently, species have evolved resistance to these insecticides via receptor site mutations with unknown impacts to their fitness. The goal of this study was to determine if pyrethroid-resistant *Hyalella azteca* populations (Escondido and Mosher) exhibited disrupted phototaxis compared to a non-resistant population. We tested phototactic response to determine the sensitivity of *H. azteca* to chemicals that cause neurobehavioral impacts. *H. azteca* were exposed to non-dosed, permethrin (pyrethroid), and fipronil (phenylpyrazole) treatments 24 h before assessing phototaxis using light stimulus. Each population was genotyped for mutations in the voltage-gated sodium channel (VGSC) and the γ-aminobutyric acid (GABA) receptor. Non-resistant *H. azteca* had a faster response to light stimulus than resistant populations following the non-dosed and fipronil-dosed exposures. When exposed to pyrethroids, the Escondido population responded more rapidly than the non-resistant population. Genotyping results revealed all populations were wild-type for *rdl*, the gene encoding the GABA-gated chloride channel and the target for phenylpyrazole insecticides. In addition, the Mosher population shifted from homozygous for the L925I substitution in the VGSC gene (*vgsc*) to a relatively weaker I936F for pyrethroid resistance. The exact cause in the reduction in negative phototaxis and general fitness is unknown but may stem from reduced protein efficiency from the *vgsc* mutations or possible results of genetic bottlenecking, hence reducing genetic diversity. Benefits of adaptation may be limited to improved survivorship under harsh conditions of insecticide exposure but at a cost to overall fitness including phototactic behavior.

**Supplementary Information:**

The online version contains supplementary material available at 10.1007/s10646-026-03084-x.

## Introduction

Pyrethroids are a class of insecticides that have seen increased use following the phase-out of organophosphate insecticides (Lao et al. 2012). Due to their low toxicity in mammals, pyrethroids are extensively used in agriculture and urban areas for pest control (Heim et al. 2018; Schulz et al. 2021), which can result in contamination of waterways via stormwater runoff, reaching concentrations that are highly toxic to aquatic invertebrates (Weston et al. 2013, 2019). *Hyalella azteca* is an epibenthic aquatic amphipod that is extremely sensitive to pyrethroid insecticides (Weston et al. 2013; Clark et al. 2015; Heim et al. 2018; Sever et al. 2020), and it has been subject to selective pressure due to frequent exposure to pyrethroid insecticides.

Several studies have shown that the development of pyrethroid resistance in *H. azteca* is due to a single amino acid substitution in the voltage-gated sodium channel (VGSC) gene (*vgsc*) at either of three different loci: M918, L925, or I936 (Weston et al. 2013; Major et al. 2018, 2022). These substitutions in the *vgsc* result in a conformational change to the VGSC receptor site, which prevents pyrethroids from binding as readily (Rinkevich et al. 2013). However, fitness costs of this type of *vgsc* mutation are known to affect the performance of the VGSC via reduced neuronal excitability (Lee et al. 1999; Zhao et al. 2000). *H. azteca* with the *vgsc* mutation were shown to exhibit decreased thermal tolerance, reduced offspring survival in higher salinities (Fulton et al. 2021) and have heightened sensitivity to other chemical stressors (Heim et al. 2018). It is unclear if these impacts are directly related to structural changes in the VGSC caused by the resistance mutations or due to reduced genetic diversity and increased homozygosity in the resistant populations.

Natural populations of *H. azteca* are commonly exposed to a myriad of chemical stressors simultaneously as frequently seen in the San Franscisco Bay-Delta region of California, USA (Weston et al. 2019). Both permethrin and fipronil have been detected in the San Francisco Bay-Delta (Weston and Lydy 2010; Weston et al. 2015; Fong et al. 2016), often at concentrations that can cause impairment in *H. azteca* (Weston et al. 2015). Therefore, pyrethroid-resistant *H. azteca* also may be more susceptible to other co-occurring insecticides like fipronil, a phenylpyrazole insecticide, due to increased fitness costs observed previously. Alternatively, it is possible that pyrethroid-resistant populations may develop cross-resistance to other compounds with similar modes of action (Brengues et al. 2003) or through mobilization of detoxification pathways (Yunta et al. 2019), decreasing sensitivity to other stressors despite resistance related fitness costs. Phenylpyrazole insecticides are also commonly used as a pest management tool (Bonmatin et al. 2015), often entering waterways through runoff (Weston et al. 2013; Budd et al. 2015) where phenylpyrazoles can reach concentrations that are toxic to non-target aquatic invertebrates such as *H. azteca* (Liu et al. 2024). Considering potential for the co-occurrence of fipronil with permethrin in aquatic systems, it is important to understand how this insecticide may be affecting *H*. *azteca* fitness, including behavioral responses.

While *H. azteca* displays a high degree of sensitivity to pyrethroid insecticides (Weston and Jackson 2009; Ranatunga et al. 2023), they are more tolerant to phenylpyrazole insecticides compared with other invertebrates (Weston and Lydy 2014; Poynton et al. 2026). Phenylpyrazole insecticides target the γ-aminobutyric acid (GABA) receptor, first identified as resistance to dieldrin (*rdl*), which is a ligand-gated chloride channel and neurotransmitter receptor (Remnant et al. 2014). The increased tolerances of *H. azteca* may be related to the presence of a serine amino acid in the 301 position of the *rdl* GABA receptor, found recently in lab and field populations of *H. azteca* (Poynton et al. 2026). What appears to be the wild-type genotype for *H. azteca* (i.e., S301) is comparable to a resistance mutation found in some insect pests (Buckingham et al. 2005). Using genotyping assays to confirm the presence of the S301 genotype in the laboratory populations of the current study will be important to understand how fipronil is affecting phototactic response in both resistant and non-resistant populations of *H. azteca.*

Behavioral bioassays are part of a suite of sublethal assays gaining popularity in ecotoxicology (Legradi et al. 2018; Ford et al. 2021; Soose et al. 2023). Assays which measure animal responses to light, or phototactic responses, have been used in genetic studies (Busto et al. 1999; Emran et al. 2008) and in ecotoxicology to characterize neurobehavior responses to chemicals (Michels et al. 2000; MacPhail et al. 2009; Sancho et al. 2025), including in amphipods (Kohler et al. 2018; Soose et al. 2023). Amphipods exhibit negative phototaxis, or movement away from light (Kohler et al. 2018), likely as an avoidance behavior since low light reduces the perception of predation risk (De Robertis 2002; Jermacz and Kobak 2023). Sudden light stimulus may be interpreted as a reduction in cover, triggering behaviors that cause amphipods to seek out shaded areas. Understanding possible alterations in phototaxis may provide further insight into the fitness costs of pyrethroid resistance.

The main objectives of our study were to determine if pyrethroid-resistant populations of *H. azteca* differed in their phototactic response compared to a non-resistant population under three different exposure scenarios: a non-dosed control test, an exposure to permethrin, and an exposure to fipronil and to measure dissimilarities in their *vgsc* and *rdl* genes. We hypothesize that the phototactic behavior of the resistant populations will be less affected under different exposure scenarios involving contaminants when compared to the non-resistant population due to the presence of the *vgsc* mutation in resistant *H. azteca*. Conversely, we expect the non-resistant population to display higher phototactic responses than the resistant populations during the non-dosed control test from the lack of fitness costs of the *vgsc* mutation in non-resistant *H. azteca.* We developed and tested a simple phototaxis response bioassay to determine the sensitivity of *H. azteca* to chemicals that cause neurobehavioral impacts. Established genotyping assays were performed on a subset of each population. This was to measure the occurrence of resistance mutations for both pyrethroids and phenylpyrazoles in each population of *H. azteca* and whether mutations to the *vgsc* and *rdl* relate to observed alterations in the phototactic behavior of resistant populations.

## Materials & methods

### Test organisms

Three populations of *H. azteca* were used for the current study: one non-resistant population and two pyrethroid-resistant populations (Mosher and Escondido). The non-resistant population was obtained from cultures managed by the U.S. EPA laboratory (Duluth, MN, USA) in 2001. The Mosher population was collected from Mosher Slough in Stockton, California in 2013 and the Escondido population was taken from Escondido Creek in San Diego, California in 2017. *H. azteca* exists as a species complex across North America and clades are distinguishable through cytochrome oxidase I (COI) sequencing (Major et al. 2018). Individuals from the Mosher lab population belong to clade D, while the Escondido and non-resistant populations belong to clade C (Heim et al. 2018; Sever et al. 2020). Despite belonging to distinct clades, previous work has shown that the presence of resistance mutations, and not clade designation has the greatest impact on sensitivity to insecticides (Heim et al. 2018; Major et al. 2018; Sever et al. 2020; Gamble et al. 2023). Methods for culturing the *H. azteca* populations followed standard U.S. EPA (2000) protocols. Further details can be found in the Supplemental Materials (SM).

### Phototactic response bioassays

Prior to the phototactic response bioassays, *H. azteca* were exposed to three different treatments for 24 h. Exposure treatments included 20 ng/L permethrin (*n* = 12), 1400 ng/L fipronil (*n* = 12), and solvent controls (*n* = 12) in moderately hard water (MHW) spiked with < 0.01% acetone (U.S. EPA, 2000). These exposure concentrations were determined from the literature and previous testing in our lab (Ma 2006; Weston and Lydy 2014; Sever et al. 2020). Exposure water for the pre-exposure tests was prepared in bulk using 4 L Erlenmeyer flasks and stirred for 20 min before 500 mL of spiked water was allocated to twelve 600 mL beakers for each target analyte (*n* = 36). Adult *H. azteca* were selected for each population using a 1-mm sieve to separate them from smaller juveniles. Ten *H. azteca* were placed into each spiked beaker, and a 6.45 sq. cm piece of Nitex screen (110 mesh) was added to serve as substrate during the exposure period. Beakers were then placed into a Model 818 incubator at 25 °C for 24 h using a 16:8 light: dark cycle (ThermoFisher Scientific, Waltham, MA, USA).

**Fig. 1:**
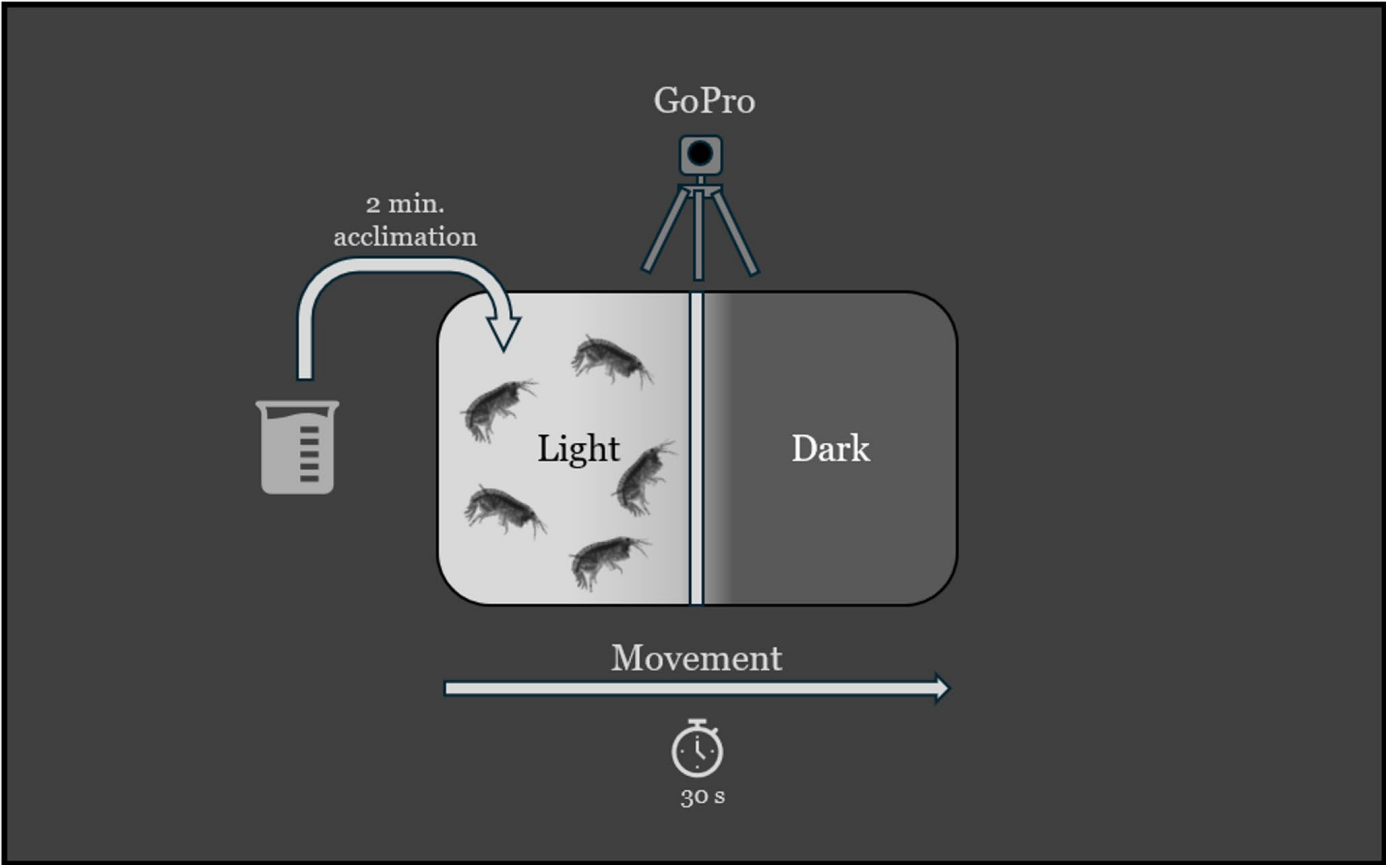
Graphical illustration of testing set-up. *Hyalella azteca* were placed on the left side of the dish to acclimate in darkness for two minutes, after which the middle barrier was removed, and the left side of the dish was illuminated. Avoidance behavior was measured for 30 s following illumination

After the 24 h exposure period, the beakers were removed from the incubator and *H. azteca* were transferred to a beaker filled with undosed MHW using a mesh screen. The *H. azteca* were then transferred to a square glass dish (19 cm x 0.25 cm x 6 cm) fitted with a plastic barrier that divided the dish into two. The dish was then placed on a light table, modified with black card stock covering half of the table to prevent illumination on one side (Fig. 1). *H. azteca* underwent a 2 min acclimation period with the plastic barrier in place preventing them from moving to the side of the light table covered with black card stock. Once acclimation had finished, *H. azteca* avoidance behavior was assessed by turning on the light table while simultaneously removing the barrier from the dish, allowing movement to the other half of the dish on the unilluminated side of the light table. The avoidance assessment was monitored for 30 s, recording the proportion of animals on the light and dark side of the dish at the end of the interval. The number of *H. azteca* on the dark side of the dish at the end of the testing duration was defined as dark side *H. azteca* (DSH) or percent negative phototaxis. Thirty seconds were chosen due to significant increase in swimming velocity within the first 30 s of exposure to sudden light stimulus (Bossus et al. 2014). Footage of each phototactic response bioassay was recorded using a Hero 7 GoPro (San Mateo, CA, USA) camera for later review.

### Genotyping methods

To determine if the *H. azteca* populations used in the current study retained their resistance mutations to pyrethroids or possessed resistant mutations for phenylpyrazole insecticides, individuals from the *H. azteca* lab cultures were genotyped at the *vgsc* and *rdl* loci based on genotyping assays developed previously (Major et al. 2018; Poynton et al. 2026). Individuals were randomly selected from each culture and placed in 95% ethanol. Genomic DNA (gDNA) was isolated from individuals using an Enzymax gDNA isolation kit (Lexington, KY, USA) using the manufacturer’s protocol with modifications. Animals were placed in “ACL buffer” with proteinase K and homogenized in a Qiagen tissue lyser for 20 min at 50 oscillations/s using a 3.2 mm stainless steel bead (BioSpec, Bartlesville, OK, USA). Samples were incubated at 55 °C overnight, then at 70 °C for 10 min before proceeding with on column gDNA purification. gDNA was isolated from individuals using the Enzymax gDNA isolation kit with modification described above. Primers were designed in a previous study (Poynton et al. 2026) to span areas of the genes where resistance mutations were previously documented in *H. azteca* or pest insects (i.e., Dong et al. 2014; Remnant et al. 2014). Partial gene segments were amplified with Phusion Plus PCR Master Mix (ThermoFisher, Waltham, MA, USA), 1 µM of each primer (Table 1), and 100 ng of template DNA using the following program: 98 °C for 30 s, 35 cycles of 98 °C for 10 s, melting temperature (see Table 1) for 30 s, and extension for 72 °C for 30 s, with a final extension at 72 °C for 10 min. PCR products were visualized on a 1.5% agarose gel and sequenced using the forward (*rdl*) or reverse primer (*vgsc*) at Quintara Bio (Boston, MA, USA). Sequences were trimmed of low-quality bases and translated to the amino acid sequence. Sequences were inspected manually for the presence of resistance mutations, and we referred to chromatographs to determine the potential for heterozygosity at the *vgsc* and *rdl* loci.

**Table 1:** Primer sequencing for *vgsc* and *rdl* genotyping

Gene	Primer sequences	Melting temp	Product size
*vgsc*	For: AGGGTGTTCAAGCTCGCTAA Rev: ACATGCTCTCGATCCACTCC	64	578 bp
*rdl*	For: CCACTCCCGCGCGCGT Rev: CAAGCAGCGAGGCGAAGACC	64	120 bp

### Toxicity bioassays

The continued presence of pyrethroid resistance in the Escondido and Mosher populations was confirmed by using acute toxicity bioassays. Individuals (10 per replicate, 3 replicates per level) were exposed to aqueous permethrin in 5 levels (nominal concentrations: 250, 500, 1000, 2000, and 4000 ng/L plus solvent control and a negative control) at 25 °C for 96 h using a 16:8 light: dark cycle. After 96 h, surviving individuals were enumerated, and the LC_50_ (the median concentration that causes lethality in 50% of the test population) was estimated using R Studio (R Core Team 2022) with the Probit method in the ecotoxicology package (Gama 2015).

### Quantification of pesticide concentrations in the water

Information on chemicals and materials used are detailed in the SM. Permethrin and fipronil were extracted from water samples from the pre-exposure tests using a solid phase extraction method (Wang et al. 2009) adapted from U.S. EPA method 525.1 (U.S. EPA 1991). Triplicate 500 mL composite water samples for each treatment were collected from bulk spikes at time zero and stored at 4 °C with 1% methanol (% v/v) as a keeper solvent. A stability test was conducted to assess any potential loss of pesticides during these pre-exposure tests. See the SM for additional information on the chemical extractions, quality assurance/quality control (QA/QC) and stability test methods. Extractions were performed at time zero for all treatments.

Analysis of pesticide concentrations in each water sample was performed using an Agilent Technologies 7890B gas chromatography (GC) system (Agilent Technologies, Palo Alto, CA, USA) equipped with a HP-5ms (30 m x 0.25 mm and 25 μm) Ultra Inert column and a 5975 C Inert XL mass spectrometry (MS) equipped with a Triple Axis Detector (Santa Clara, CA, USA) using negative chemical ionization mode. Methane was used as a reagent gas. Extracts were injected onto the column using a 1.0 µL injection in pulsed splitless mode (3.45 × 10^5^ Pa for 0.75 min) at 250 °C then separated via helium as a carrier gas (1 mL/min). Oven temperatures were ramped from an initial temperature of 50 °C to 200 °C, with a rate of 20 °C/min. Once at 200 °C, the rate was increased by 10 °C/min until 295 °C was reached, for a total run time of 27 min. The MS interface was set to a temperature of 300 °C, source at 150 °C, and quadrupole at 150 °C. The instrument was validated using a 50-ppb mid-standard check run every 8 sample to ensure accuracy was within 80–120%. If checks were outside the acceptable range, corresponding samples were rerun after performing maintenance on the instrument.

### Statistical analysis

All statistical analyses were conducted using RStudio version 1.3.1093 (RStudio Team, 2020) operating with R version 4.1.2 (R Core Team, 2022). A two-way ANOVA (α = 0.05) and subsequent tests for simple effects utilizing packages ‘phia’ (Rosario-Martinez et al. 2025), ‘multcomp’ (Hothorn et al. 2008), and ‘emmeans’ (Lenth et al. 2025) were used to analyze the effects of population, treatment, and their interaction on the percent of *H. azteca* displaying negative phototaxis at the end of the bioassay. Differences in the measured permethrin and fipronil concentrations among populations were measured using one-way ANOVA (α = 0.05).

## Results

### Pesticide concentrations in pre-exposure water

Analysis of the water from the non-dosed tests showed no detectable (< reporting limit) concentrations of permethrin and fipronil. Sublethal permethrin water concentrations (average ± standard deviation) measured at the start of the exposure period were not significantly different among the populations and averaged 15.04 ± 1.79 ng/L for Escondido, 16.34 ± 2.21 ng/L for Mosher, and 21.90 ± 0.50 ng/L for the non-resistant population. Approximately 25% of the permethrin concentration was expected to be lost throughout the test duration based on the stability study results, so the time averaged exposure concentrations were 13.16 ng/L, 14.30 ng/L, and 16.43 ng/L. Sublethal fipronil water concentrations (average ± standard deviation) at the start of the pre-exposure period were not statistically different and were 1397 ± 178 ng/L for Escondido, 1465 ± 58 ng/L for Mosher, and 1262 ± 92 ng/L for the non-resistant population. The preliminary stability test suggested approximately 31% of fipronil was expected to be lost during the test, so the time averaged exposure concentrations were 1181 ng/L, 1282 ng/L, and 1067 ng/L.

The nominal 96 h LC_50_ values (95% confidence intervals) for Mosher and Escondido were 230 (35–444) ng/L and 2140 (1480–3780) ng/L permethrin, respectively for tests confirming the continued presence of pyrethroid resistance in the Escondido and Mosher populations. For the non-resistant population, a screening bioassay found a similar LC_50_ as previous testing of this population over time which was 31.2 ng/L (Muggelberg et al. 2017; Sever et al. 2020).

### Characterization of resistance in *H. azteca* populations

Genotyping at the *vgsc* loci showed the three populations maintained consistent *vgsc* genotype frequencies from our previous studies (Gamble et al. 2023). The non-resistant population was homozygous wild type at all loci, while the Escondido population harbored a single nucleotide mutation resulting in the L925I substitution (Table 2, Table **S1**). The genotypes for the Mosher population were mixed, resulting in either a phenylalanine substitution at I936, isoleucine and valine substitutions at L925, or both. Half of the individuals were heterozygous for these two substitutions, but none of the Mosher individuals had a wild-type genotype for *vgsc* (Table 2, Table S1).

**Table 2:** Genotype frequencies for the *vgsc* and *rdl* locus in populations used in the current study. The percentage of individuals (*n* = 6–8) with the given homozygous or heterozygous genotypes is given for each population. See Table S1 for detailed genotypes for each individual

Population	*vgsc*					*rdl*
	Homozygous			Heterozygous		Homozygous
	L925 (wt)/ I936 (wt)	L925I/ I936 (wt)	L925 (wt)/ I936F	L925V/ I936F^a^	L925I/ I936F^a^	S301 (wt)
Non-resistant	100%	na	na	na	na	100%
Escondido	na	100%	na	na	na	100%
Mosher	na	25%	25%	12.5%	37.5%	100%

^a^For L925I/I936F and L925V/I936F heterozygous substitutions, the assay used in the current study cannot distinguish which allele the mutations are on. In previous work we have not found any individuals with both resistant mutations on a single allele, and therefore, our best approximation is that they are heterozygous for those two mutations. na = not applicable

Sequencing of the *rdl* locus in all populations revealed that all populations had wild-type genotype resulting in a serine at position 301. While this is the wild-type genotype for crustaceans in the class Malacostraca (Poynton et al. 2026), this genotype resembles the A301S substitution in fipronil-resistant insects (Buckingham et al. 2005).

### Phototaxis response

**Fig. 2:**
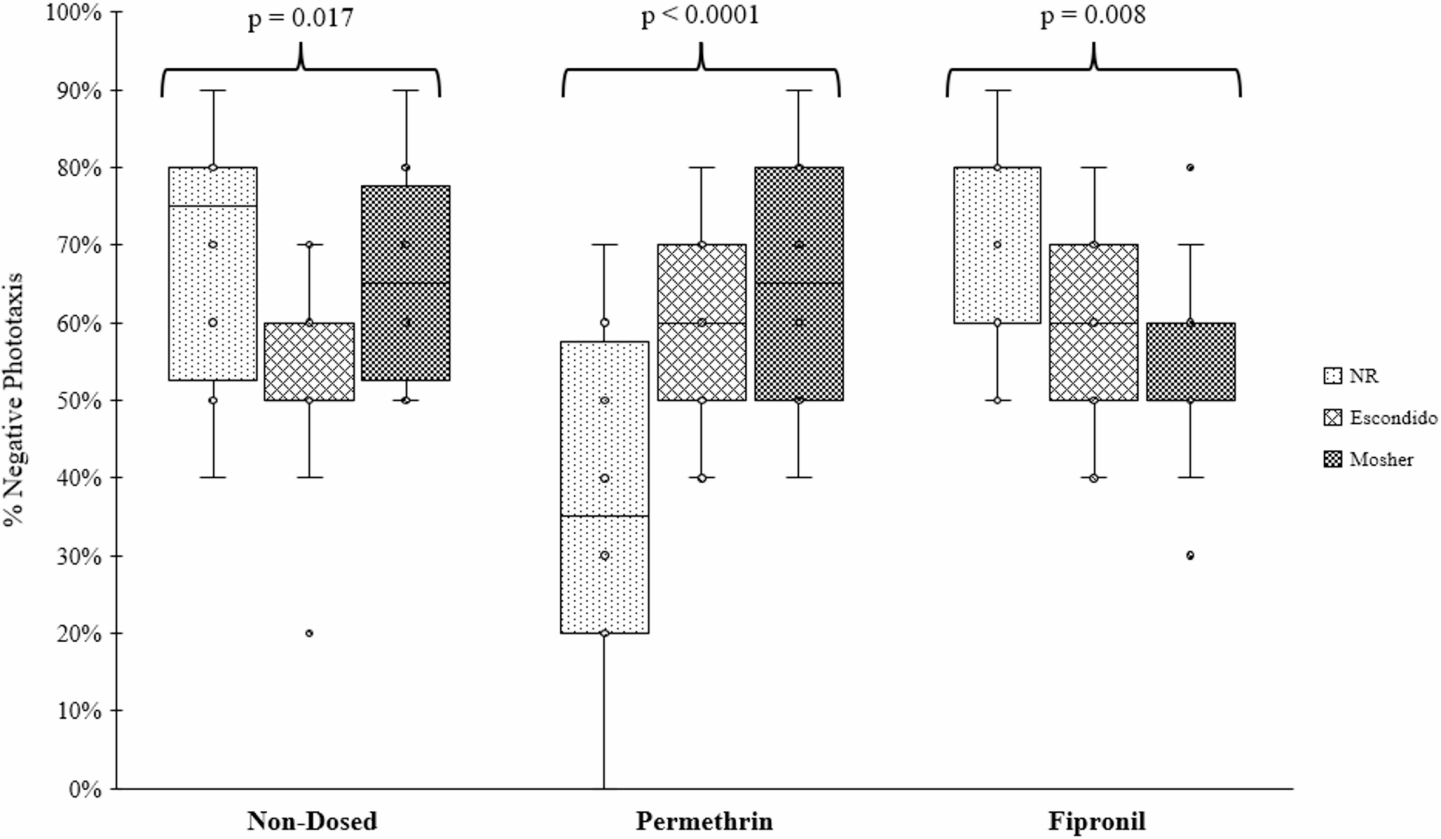
Comparing altered phototactic response in *Hyalella azteca* using a two-way ANOVA and subsequent test for simple effects. Significance indicates the simple effects between the interaction fixed for population across treatment after 30 s. NR = non-resistant population

Our behavioral phototaxis assay revealed differences in the speed and ability of *H. azteca* populations to respond to light stimulus and move toward the dark side of the assay dish. Results from the two-way ANOVA showed that treatment and population had significant interactions on altered phototaxis represented as percent negative phototaxis at 30s during the trials (F = 10.25, *p* < 0.0001), so the following interpretations are limited to the interactions between population and treatment (Fig. 2). The different *H. azteca* populations showed significantly altered phototactic responses within treatment groups (non-dosed [F = 4.28, *p* = 0.017], permethrin [F = 12.52, *p* < 0.0001], and fipronil [F = 5.08, *p* = 0.008]). The non-resistant population had the highest median percent negative phototaxis in both the non-dosed (median = 75% negative phototaxis) and fipronil treatments (80% negative phototaxis) compared to the two resistant populations at the end of the 30 s test, but the lowest when exposed to permethrin (35% negative phototaxis). In the non-dosed treatment, the Escondido population had the lowest median negative phototaxis of 50%, where in both the permethrin and fipronil treatments, the median negative phototactic response for Escondido fell between both Mosher and non-resistant. Of the Mosher population responses, fipronil had the lowest median negative phototactic response (50% negative phototaxis), permethrin had the highest (65% negative phototaxis), and the non-dosed treatment had second highest. For the effects of the different treatments by populations, only the non-resistant population showed significant changes in negative phototaxis (F = 20.96, *p* < 0.0001; Fig.**S1**). The phototactic response for the non-resistant population remained consistent between the fipronil and non-dosed treatment, but a notable decrease in negative phototaxis was observed when exposed to permethrin. Both the Escondido (F = 0.52, *p* = 0.5970) and Mosher (F = 2.86, *p* = 0.0619) treatment contrasts were not significant (Fig. S1).

## Discussion

### Phototactic behavior is a sensitive indicator of toxicity in *H. azteca*

The present study describes a phototaxis bioassay to determine the sensitivity of *H. azteca* to chemicals that cause neurobehavioral impacts. We exposed *H. azteca* to two different insecticides for 24 h and were able to observe decreases in their phototactic response after 30 s using a simple behavioral assay, and a single, easy-to-record endpoint. In the non-resistant population, we observed a decrease in phototactic response in the permethrin treatment as shown by fewer DSH. Negative phototaxis is an essential behavior in amphipods that reduces their risk of predation (Kohler et al. 2018; Jermacz and Kobak 2023). Like other behavioral assays, the decrease in negative phototaxis from contaminant impairment recorded in our assay translates to the reduction of an organism’s ability to deal with external stress, which may ultimately affect population dynamics (Ford et al. 2021). Phototactic response is driven by multiple factors including sensory perception and locomotion (Magester et al. 2021; Rajewicz et al. 2023), where reduction in negative phototaxis from contaminant impairment may decrease the ability of affected amphipods to respond to sudden light stimulus, possibly increasing the chance of predation. For aquatic amphipods like *H. azteca* who are reliant on negative phototaxis for survival (Kohler et al. 2018; Jermacz and Kobak 2023), testing for altered phototaxis should be included as an endpoint alongside other established endpoints of behavioral responses (e.g., speed and movement distance). Quantifying the effects of contaminant exposure on the disruption of negative phototaxis is important in understanding contaminant sensitivity at sublethal levels, and for our purposes, quantify potential fitness costs of resistant mutations.

### Phototactic response correlated with differences in population and genotype

Differences in the response of each resistant and non-resistant population to insecticides were detected. When exposed to a pesticide stressor via permethrin treatments, resistant populations showcased more negative phototactic behavior compared to the non-resistant population. The response from both the Escondido and Mosher populations was similar with 60 to 70% of these amphipods exhibiting negative phototaxis in contrast to less than 40% in the non-resistant population. Resistant *H. azteca* populations have decreased pyrethroid sensitivity due to the reduced pyrethroid binding to the VGSC with either the L925I or I936F substitution (Rinkevich et al. 2013; Weston et al. 2013). The lack of mutations in the *vsgc* of the non-resistant population resulted in higher pyrethroid sensitivity, thus reduced negative phototaxis when exposed to permethrin compared to its resistant counterparts. A similar phenomenon was also demonstrated in lab cultured populations of *Daphnia magna*, where exposure to the pyrethroid lambda-cyhalothrin and fipronil disrupted swimming behavior, resulting in shorter distances travelled and slower swimming speeds (Bownik et al. 2019; Bownik and Szabelak 2021).

### The presence of resistance mutations impairs the phototactic response in absence of pyrethroid insecticides

While the presence of resistance mutations provided a protective effect to Escondido and Mosher populations in the permethrin exposures, in the absence of permethrin, the Escondido and Mosher population trended to have a reduced phototactic response compared to the non-resistant population, with the Escondido population having the lowest median negative phototaxis. The dissimilarity in the severity of altered phototactic response between the two resistant populations, as well as the observed disruption in negative phototaxis compared to the non-resistant population suggest possible fitness impacts to the animals harboring resistance mutations. Different *vgsc* mutations may have various associated fitness costs. In the Escondido population all the individuals sampled in this and previous studies (Sever et al. 2020; Gamble et al. 2023) contain a *vgsc* mutation resulting in a L925I substitution, while the Mosher population contains a mix of individuals with both L925I and I936F genotypes present. In laboratory culture, the allele frequency of the Mosher population has shifted over time (Table S2). *H. azteca* originally sampled from Mosher Slough and cultured in our laboratory were mostly homozygous for the L925I substitution when sampled between 2014 and 2018. However, as of 2023, genotyping assays showed that the L925I substitution has become much less common in our Mosher lab population, while the relatively less potent I936F substitution was found in 90% of the sequenced animals (Gamble et al. 2023). This is supported by our most recent acute toxicity test, showing that the Mosher population has a much lower nominal permethrin LC_50_ (232 ng/L) compared to the Escondido population (LC_50_ = 2140 ng/L). The I936F substitution provides only a 4-fold increase in tolerance to pyrethroids (Major et al. 2022) compared to the 266-fold resistance from the L925I mutation (Heim et al. 2018; Major et al. 2018; Sever et al. 2020), but this would be of no consequence to a population being cultured in the absence of pyrethroids. Changes in the *vgsc* mutation frequency from L925I to I936F in the Mosher population may indicate lowered fitness costs of the I936F or may be an indication of genetic drift within the small, finite laboratory population.

In addition, the pyrethroid-resistant populations also displayed decreased phototaxis response in the fipronil exposures compared to the non-resistant population. Like populations of *H. azteca* sampled in the Sacramento River watershed of California (Poynton et al. 2026), all the individuals in the current study had the same genotype for the fipronil target gene, *rdl* (e.g., S301). The S301 genotype differs from the insect *rdl* gene but is found in most crustaceans and provides increased tolerance to phenylpyrazole insecticides (Poynton et al. 2026). In the fipronil treatments, the pyrethroid-resistant populations had fewer displays of negative phototaxis, demonstrating that the pyrethroid-resistant populations have decreased negative phototaxis compared to the non-resistant population. The resistant populations have an advantage in the presence of pyrethroid insecticides. In the absence of pyrethroids, whether other stressors are present or not, they display a disadvantage relative to the non-resistant population. It has been previously observed that other environmental stressors like increased temperature and salinity can have significant interactions on the uptake and elimination of contaminants in both resistant and non-resistant populations of *H. azteca*. Biotransformation rates were elevated at higher temperatures for Mosher and Escondido *H. azteca*, and Escondido *H. azteca* demonstrated higher bioconcentration factors at elevated salinities (Derby et al. 2022). This suggests additional costs may be associated with resistance under future climate scenarios.

This study establishes that pyrethroid-resistant populations experience reduced phototactic response when exposed to uncontaminated environments or in the presence of other contaminants, where the severity of the effects is correlated to the population’s genotype. However, more evidence is needed to properly understand how genotypic changes relate to the reduction of fitness (e.g., altered phototactic response; reduced reproductive rates, decreased thermal tolerance, reduced neuronal excitability). Possible explanations to the reduction in fitness may be that mutations to the *vgsc* resistance results in reduced efficiency of the VGSC protein (Lee et al. 1999; Zhao et al. 2000; Heim et al. 2018; Fulton et al. 2021) or genetic bottlenecking. Voltage clamp experiments performed with *vgsc* constructs harboring different resistance mutations reveal differences in the electrophysiological properties of these VGSC proteins (Lee et al. 1999; Zhao et al. 2000; Usherwood et al. 2007). In a study on the gating properties of several different mutant VGSC proteins, differences in the voltage-dependent activation and inactivation potentials across channels were observed (Usherwood et al. 2007). Different substitutions impact the sodium channel gating function by shifting the voltage-dependent activation and inactivation of the channel, potentially reducing the excitability of the neuron, and impacting neuromuscular function proteins (Lee et al. 1999; Zhao et al. 2000). While mutations to the VGSC have been shown to impact neuron excitability and may be responsible for different fitness costs across individuals with different genotypes, the exact impact on *H. azteca* with L925I or I936F mutations is not known.

Alternatively, the fitness impacts observed in the Mosher and Escondido populations could be a result of a genetic bottlenecking when the populations developed resistance to pyrethroids. Severe population decline from exposure to runoff containing lethal concentrations of pyrethroids may result in evolutionary rescue, the rapid evolution of a population from individuals possessing a resistance adaptation post the selection event (Wahl and Campos 2023; Draghi et al. 2024; Orr and Unckless 2014; Stewart et al. 2017). While evolutionary rescue prevents population extinction, genetic bottlenecking may occur because of a limited gene pool (Van Straalen and Timmermans 2002; Stewart et al. 2017), thus reducing overall fitness via increased genetic homogeneity and inbreeding depression. Resistant *H. azteca* have reduced reproductive rates (Heim et al. 2018) and decreased thermal and salinity tolerance (Heim et al. 2018; Fulton et al. 2021), which can impact population fitness. Studies involving other arthropod species have shown that resistant animals have a reduced ability to detect predator cues, like alarm pheromones in aphids (Foster et al. 2005), or display behavioral resistance (Chandi and Singh 2017; Hubbard and Murillo 2024), which can be defined as an action that has evolved in response to the selective pressures exerted by a toxicant and enhances the ability of a population to avoid the lethal effects of that exposure (Lockwood et al. 1984). Similarly, increased homogeneity and reduced genetic diversity may be responsible for the decreased phototactic response that we observed in Mosher and Escondido populations.

More evidence is needed to properly understand how genotypic changes relate to the reduction of fitness such as the assessment of neutral genetic markers for population genetic diversity. Including this in futures studies will help strengthen the relationship between genotypic change and altered fitness. Another limitation of the current study is the inability to distinguish whether the disruption in phototaxis originates from movement reduction, impaired light perception, or a combination of factors. Other tests can be done in conjunction with the current phototaxis bioassay, such as monitoring swimming behavior or calculating movement rates to broaden our understanding of the potential implications to developing pesticide resistance. Possible mating interactions could have interfered with movement in the testing chambers, as sex ratio was not controlled during this experiment. Future studies would benefit from controlled sex ratios within each testing group to eliminate potential interference. Additionally, our data was limited to just one non-resistant population, and one population with each of VGSC substitutions. Genetic drift may occur in small laboratory populations that could influence phototaxis and other behaviors. Including additional populations in future studies will provide a stronger relationship between phototaxis and pyrethroid resistance.

## Conclusions

A new phototaxis response bioassay was tested to determine the sensitivity of *H. azteca* to chemicals that cause neurobehavioral impacts. When *H. azteca* were exposed to permethrin for 24 h prior to the bioassay we found that the pyrethroid resistant populations were favored and displayed more negative phototaxis, while the opposite result was found under fipronil treatments. In non-dosed treatments, the Escondido population exhibited less negative phototaxis compared to the non-resistant population, while the Mosher population fell between the Escondido and non-resistant populations. Differences between the two resistant populations may be from amino acid substitutions in the VGSC, where the Mosher population has experienced a shifted allele frequency towards the relatively less potent I936F substitution compared to the L925I/V substitution found in Escondido. Two hypotheses have been proposed to explain these results. First, the fitness costs associated with the *vgsc* resistance mutations may result directly from reduced efficiency of the VGSC protein potentially reducing the excitability of the neuron and impacting neuromuscular function. Alternatively, the fitness impacts observed in the Mosher and Escondido populations could be a result of a genetic bottleneck that occurred when the populations developed resistance to pyrethroids, thereby reducing the genetic diversity of these resistant populations which is the cause of the noted fitness costs. However, these hypotheses need additional genetic testing to support it. More research is needed to better understand the relative role each mechanism has in the phototactic response. A benefit of pyrethroid resistance in *H. azteca* is for the improved survivorship under harsh conditions of insecticide exposure, but at a cost to overall fitness via the alteration of negative phototaxis.

## Supplementary Information

Below is the link to the electronic supplementary material.

Supplementary Material 1

## Data Availability

Data generated from the project will be stored in SIU’s open-access institutional repository (OpenSIUC, http:/opensiuc.lib.siu.edu). OpenSIUC provides access to its content via any internet browser, with no user registration required.
