# Supplementary material for: Assessing phototaxis behavior in non-resistant and insecticide-resistant populations of Hyalella azteca
Source: Ecotoxicology. 2026 Apr 24;35(5):103. doi: 10.1007/s10646-026-03084-x (PMC13109139; doi:10.1007/s10646-026-03084-x)
Supplement: Supplementary file 1 — Supplementary Material 1 [file 10646_2026_3084_MOESM1_ESM.docx]

**Assessing phototaxis behavior in non-resistant and**

**insecticide-resistant populations of *Hyalella azteca***

Nick Hettel^a,b^, Cristina G.B. La^a,b^, Helen Poynton^c^, Giovanni S. Molinari^a,b^, Kara E. Huff Hartz^a,b^, and Michael J. Lydy^a,b*^

^a^ Center for Fisheries, Aquaculture and Aquatic Sciences and Department of Zoology, Southern Illinois University, Carbondale, IL, 62901, USA

^b^ Department of Zoology, Southern Illinois University, Carbondale, IL, 62901, USA

^c^ School for the Environment; University of Massachusetts; Boston, Massachusetts, 02125, USA

***Corresponding Author**

Michael J. Lydy ([mlydy@siu.edu](mailto:mlydy@siu.edu)); 1-618-453-4091

Center for Fisheries, Aquaculture, and Aquatic Sciences; School of Biological Sciences; Southern Illinois University; Carbondale, Illinois, 62901; USA

**Supplemental Materials**

**Materials and Methods**

*Test Organisms & Culturing*

All populations of *Hyalella azteca* were cultured in 40 L glass aquarium tanks that were filled with moderately hard water (MHW) and maintained at a temperature of 23-25°C (U.S. EPA, 2000). These tanks were continually aerated and water quality was tested on a monthly basis, with the aim of maintaining parameters within the following ranges: temperature at 25°C ± 2°C, pH between 6.5 and 8.0, conductivity between 350 and 450 µS/cm, ammonia <0.1 mg/L, and dissolved oxygen greater than or equal to 4 mg/L. Conductivity and pH were measured using an Oakton waterproof 150 conductivity meter and an Oakton waterproof 150 pH meter (Oakton Instruments, Vernon Hills, IL, USA). Temperature was measured using the conductivity probe in the interest of consistency. Dissolved oxygen was measured with a Yellow Springs Incorporated (YSI) model Pro20i Professional Series dissolved oxygen meter (Yellow Springs, OH, USA). Ammonia measurements were obtained using an Aquarium Pharmaceuticals Incorporated (API) freshwater ammonia test kit (Mars Fishcare Inc., Chalfont, PA, USA). When water quality parameters were found to be out of range, a 30% water change was conducted using either deionized water, MHW, or a 1:1 mixture of both depending on conductivity and ammonia measurements.

In the cultures, the *H. azteca* feeding regimen consisted of approximately 20 mL of a Tetramin slurry, 5 mL of diatoms (*Thalassiosira weissflogii*), and 5 mL of *Selenastrum capricornutum,* all of which was administered three times each week. Sugar maple leaves (*Acer saccharum*) were also added to each tank to provide a substrate as well as an additional source of food. These leaves were presoaked for 30 days in 30 L plastic tubs, along with 300 g of canning salt (Morton Salt, Overland Park, KS, USA) to remove toxic tannins prior to their addition to the culture tanks.

*Chemical*s

The standards including decachlorobiphenyl (DCBP, surrogate) and 2,2',3,4,4',5,6,6'-octachlorobiphenyl (PCB-204) internal standard were purchased from AccuStandard. The ^13^C_12_-DCBP internal standard was purchased from Cambridge Isotope Lab Inc. (Tewksbury, MA, USA). Methanol (pesticide grade) and acetone and hexane (Optima grade) were purchased from Fisher Scientific (Hampton, NH, USA). The permethrin (isomer ratio of 46% cis and 54% trans) and fipronil stocks used in the bioassays were both made in acetone and purchased from AccuStandard (> 97% purity; New Haven, CT, USA). Hypersep 500 mg/2.8 mL C18 solid phase extract cartridges were obtained from Fisher Scientific. Acetic acid (glacial 99.59%) was sourced from Mallinckrodt (Staines-upon-Thames, UK).

*Stability test*

A preliminary stability test was conducted with permethrin and fipronil to characterize the amount of compound that may have degraded or was lost to glassware binding over the 24 h testing period. At the start of the stability test, spiked water for both compounds and solvent controls was collected as a composite sample in triplicate from each Erlenmeyer flask, for a total of 500 mL of spiked water for each compound. The time zero samples each received 5 mL of methanol to function as a keeper solvent before they were placed in a Samsung RS28A500ASR refrigerator (Samsung Electronics, Suwon-si, South Korea) at 2.8°C for 24 h. The remaining bulk spiked water was used to make triplicate samples for both compounds and solvent controls that were placed in an incubator at 25°C for 24 h. The following day, both time zero samples and the incubated samples were collected and permethrin and fipronil were extracted from the water using solid phase extraction (Wang et al. 2009).

*Quantification of pesticide concentrations in the water*

Permethrin and fipronil were extracted from water samples using a solid phase extraction method (Wang et al. 2009). Triplicate 500 mL composite water samples for each treatment were collected in an Erlenmeyer flask at the beginning of the exposure and stored at 4°C with 1% methanol by volume to function as a keeper solvent. Samples were brought to room temperature before extraction and spiked with 40 µL of 1 µg/mL surrogates in acetone. Hypersep 500 mg C18 solid phase extraction cartridges (ThermoFisher Scientific, Waltham MA, USA) were attached to a Visiprep (Millipore Sigma, Burlington MA, USA) 12 port vacuum manifold and conditioned with 3 mL of 1:1 (v/v) hexane and acetone solvent mixture, 3 mL of methanol, and 6 mL of deionized water. Water samples were then loaded onto the cartridge at a flow rate of 3-5 mL/min under vacuum and allowed to dry for 10 min to remove excess water before closing the manifold and receiving two 4 mL rinses of 1:1 hexane and acetone solvent (v/v) mixture, to be collected in the culture tubes under vacuum. The cartridges were then eluted with 3 mL of hexane under gravity and rinsed three times with hexane. The culture tubes were removed from the manifold and their contents transferred to 20 mL scintillation vials (ThermoFisher Scientific, Waltham MA, USA) before being placed on a Pierce Model 1878 Reactivap (Rockford IL, USA) to undergo a solvent exchange. Samples were then evaporated under a stream of nitrogen gas and rinsed with hexane three times until the organic layer was approximately 2 mL. Samples underwent additional cleanup using anhydrous sodium sulfate prebaked at 400°C for 4 h to remove excess water. The sample extracts were evaporated down to 0.5 mL under nitrogen and transferred to gas chromatography autosampler vials with three 0.5 mL hexane rinses, evaporating to a final volume of 0.5 mL.

Alongside the extraction of permethrin and fipronil water samples, four quality assurance/quality control (QA/QC) samples were prepared: a lab blank (LB), a matrix blank (MB), a matrix spike (MS), and a matrix spike duplicate (MSD). The LB consisted of deionized water, while the MB, MS, and MSD were made using MHW. The MS and MSD received 40 µL of 1 µg/mL surrogates and 40 µL of 1 µg/mL target analytes (permethrin and fipronil) in acetone, while the LB and MB received only 40 µL of surrogates. A spike check was prepared by adding 40 µL of surrogates and target analytes to 920 µL of hexane.

**References**

Gamble NE, Huff Hartz KE, Figuero AE, Poynton HC, Lydy MJ (2023) Development of insecticide resistance in *Hyalella azteca*. Environ Pollut 322: 121165. https://doi.org/10.1016/j.envpol.2023.121165

Heim JR, Weston DP, Major K, Poynton H, Huff Hartz KE, Lydy MJ (2018) Are there fitness costs of adaptive pyrethroid resistance in the amphipod, *Hyalella azteca*? Environ Pollut 235: 39–46. <https://doi.org/10.1016/j.envpol.2017.12.04>

Major KM, Weston DP, Lydy MJ, Wellborn GA, Poynton HC (2018) Unintentional exposure to terrestrial pesticides drives widespread and predictable evolution of resistance in freshwater crustaceans. Evol Appl 11(5): 748–761. https://doi.org/10.1111/eva.12584

Sever HC, Heim JR, Lydy VR, Fung CY, Huff Hartz KE, Giroux MS, Andrzejczyk N, Major KM, Poynton HC, Lydy MJ (2020) Recessivity of pyrethroid resistance and limited interspecies hybridization across *Hyalella* clades supports rapid and independent origins of resistance. Environ Pollut 266: 115074. https://doi.org/10.1016/j.envpol.2020.115074

U.S. EPA (2000) Methods for measuring the toxicity and bioaccumulation of sediment-associated contaminants with freshwater invertebrates.

Wang D, Weston DP, Lydy MJ (2009) Method development for the analysis of organophosphate and pyrethroid insecticides at low parts per trillion levels in water. Talanta 78(4): 1345–1351. https://doi.org/10.1016/j.talanta.2009.02.012


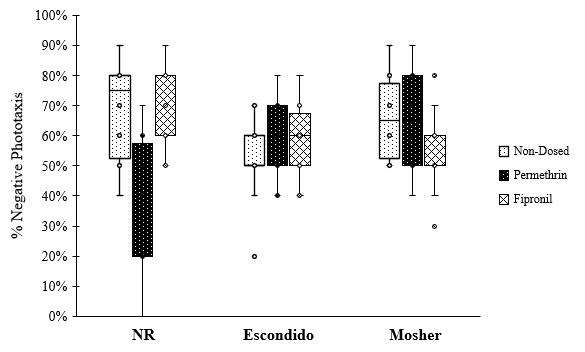


**Fig. S1.** Comparing altered phototactic response in *Hyalella azteca* using a two-way ANOVA and comparing effects of the different treatments by populations. Significance indicates the simple effects between the interaction fixed for treatments across populations after 30 s. NR = non-resistant population.

| SeqID | Clade | *rdl* S301 | *vgsc* M918 | *vgsc* L925 | *vgsc* I936 | translated *rdl* seq | translated *vgsc* seq |
| --- | --- | --- | --- | --- | --- | --- | --- |
| ESC1 | Escondido | TCC | ATG | ATC | ATC | TPARVSLGVTTVLTMTTLMSSTNAAVPK | SWPTLNLLISIMGKTVGAIGNLTFVLCIIIFIFAVMGMQLFGKN |
| ESC2 | Escondido | TCC | ATG | ATC | ATC | TPARVSLGVTTVLTMTTLMSSTNAAVPK | SWPTLNLLISIMGKTVGAIGNLTFVLCIIIFIFAVMGMQLFGKN |
| ESC3 | Escondido | TCT | ATG | ATC | ATC | TPARVSLGVTTVLTMTTLMSSTNAAVPK | SWPTLNLLISIMGKTVGAIGNLTFVLCIIIFIFAVMGMQLFGKN |
| ESC4 | Escondido | TCT | ATG | ATC | ATC | TPARVSLGVTTVLTMTTLMSSTNAAVPK | SWPTLNLLISIMGKTVGAIGNLTFVLCIIIFIFAVMGMQLFGKN |
| ESC5 | Escondido | TCC/T | ATG | ATC | ATC | TPARVSLGVTTVLTMTTLMSSTNAAVPK | SWPTLNLLISIMGKTVGAIGNLTFVLCIIIFIFAVMGMQLFGKN |
| ESC6 | Escondido | TCC | ATG | ATC | ATC | TPARVSLGVTTVLTMTTLMSSTNAAVPK | SWPTLNLLISIMGKTVGAIGNLTFVLCIIIFIFAVMGMQLFGKN |
| ESC7 | Escondido | TCC/T | ATG | ATC | ATC | TPARVSLGVTTVLTMTTLMSSTNAAVPK | SWPTLNLLISIMGKTVGAIGNLTFVLCIIIFIFAVMGMQLFGKN |
| ESC8 | Escondido | TCT | NA | NA | NA | TPARVSLGVTTVLTMTTLMSSTNAAVPK | bad sequence |
| MS1 | Mosher | TCC | ATG | G/CTC | T/ATC | TPARVSLGVTTVLTMTTLMSSTNAAVPK | SWPTLNLLISIMGKTVGA[V/L]GNLTFVLCII[I/F]FIFAVMGMQLFGKN |
| MS2 | Mosher | TCC | ATG | A/CTC | T/ATC | TPARVSLGVTTVLTMTTLMSSTNAAVPK | SWPTLNLLISIMGKTVGA[I/L]GNLTFVLCII[I/F]FIFAVMGMQLFGKN |
| MS3 | Mosher | TCC/T | ATG | A/CTC | T/ATC | TPARVSLGVTTVLTMTTLMSSTNAAVPK | SWPTLNLLISIMGKTVGA[I/L]GNLTFVLCII[F/I]FIFAVMGMQLFGKN |
| MS4 | Mosher | TCC | ATG | A/CTC | T/ATC | TPARVSLGVTTVLTMTTLMSSTNAAVPK | SWPTLNLLISIMGKTVGA[I/L]GNLTFVLCII[F/I]FIFAVMGMQLFGKN |
| MS5 | Mosher | TCC/T | ATG | CTC | TTC | TPARVSLGVTTVLTMTTLMSSTNAAVPK | SWPTLNLLISIMGKTVGALGNLTFVLCIIFFIFAVMGMQLFGKN |
| MS6 | Mosher | TCC/T | ATG | CTC | TTC | TPARVSLGVTTVLTMTTLMSSTNAAVPK | SWPTLNLLISIMGKTVGALGNLTFVLCIIFFIFAVMGMQLFGKN |
| MS7 | Mosher | TCC/T | ATG | ATC | ATC | TPARVSLGVTTVLTMTTLMSSTNAAVPK | SWPTLNLLISIMGKTVGALGNLTFVLCIIFFIFAVMGMQLFGKN |
| MS8 | Mosher | TCC | ATG | ATC | ATC | TPARVSLGVTTVLTMTTLMSSTNAAVPK | SWPTLNLLISIMGKTVGAIGNLTFVLCIIIFIFAVMGMQLFGKN |
| NR1 | US Lab | NA | ATG | CTC | ATC | bad sequence | ---------SIMGKTVGALGNLTFVLCIIIFIFAVMGMQLFGKN |
| NR2 | US Lab | TCC | ATG | CTC | ATC | TPARVSLGVTTVLTMTTLMSSTNAAVPK | ------LLISIMGKTVGALGNLTFVLCIIIFIFAVMGMQLFGKN |
| NR3 | US Lab | TCC | ATG | CTC | ATC | TPARVSLGVTTVLTMTTLMSSTNAAVPK | SWPTLNLLISIMGKTVGALGNLTFVLCIIIFIFAVMGMQLFGKN |
| NR4 | US Lab | TCC | ATG | CTC | ATC | TPARVSLGVTTVLTMTTLMSSTNAAVPK | ---------SIMGKTVGALGNLTFVLCIIIFIFAVMGMQLFGKN |
| NR5 | US Lab | TCC | NA | NA | NA | TPARVSLGVTTVLTMTTLMSSTNAAVPK | bad sequence |
| NR6 | US Lab | TCC | ATG | CTC | ATC | TPARVSLGVTTVLTMTTLMSSTNAAVPK | SWPTLNLLISIMGKTVGALGNLTFVLCIIIFIFAVMGMQLFGKN |
| NR7 | US Lab | TCC | NA | NA | NA | TPARVSLGVTTVLTMTTLMSSTNAAVPK | bad sequence |
| NR8 | US Lab | NA | ATG | CTC | ATC | bad sequence | ---------SIMGKTVGALGNLTFVLCIIIFIFAVMGMQLFGKN |
|  |  |  |  |  |  |  |  |
|  | KEY |  | KEY | KEY | KEY |  |  |
|  | TCC = S |  | ATG=M | ATC=I | ATC = I |  |  |
|  | TCT = S |  |  | CTC=L | TTC = F |  |  |
|  |  |  |  | GTC=V |  |  |  |

**Table S1**. Genotypes for individual *Hyalella azteca* analyzed in this study. Eight individuals from each population were preserved in 95% ethanol, gDNA was extracted, and partial gene sequences were amplified using *H. azteca* specific primers. The key refers to codon sequences that result in specific amino acids in the translated sequence. Sequences with multiple amino acids at a position (e.g., [I/F]) refer to heterozygotes.

**Table S2.** Pyrethroid sensitivity and genotypes of *Hyalella azteca* from the Mosher population over time. While the freshly collected Mosher population consisted of both animals from clades B and D, only clade D was ever found in the lab population or during the collection in 2016. Genotypes shifted in the Mosher lab populations between 2018 and 2022. *Sensitivity was determined by 96-h toxicity tests to the pyrethroid insecticide shown in parenthesis. **denotes wild collected *H. azteca* from Mosher Slough.

| Date | Clade | Genotype | Sensitivity (LC_50_)* | Reference |
| --- | --- | --- | --- | --- |
| Oct. 2015** | B | 94% L925I | 99 ng/L^a^ | Major et al. (2018) |
|  | D | 87% L925I, 13% L925V |  |  |
| Nov. 2016** | D | 70% L925I, 30% L925I/L925V | 3310 ng/L^b^ | Heim et al. (2018) |
| Dec. 2016** |  |  | 1803 ng/L^b^ |  |
| July 2015 | D | 85% L926I, 15% L925I/L925V | 1144 ng/L^b^ | Heim et al. (2018) |
| Feb. 2016 |  |  | 1668 ng/L |  |
| Oct. 2016 | D | 90% L925I, 10% L925I/L925V | 1409 ng/L^b^ | Sever et al. (2020) |
| Jan. 2018 |  |  | 1120 ng/L^b^ |  |
| Feb. 2022 | ND | 10% L925I, 50% L925I/I936F,40% I936F | 109 ng/L^c^ | Gamble et al. (2023) |
|  |  |  |  |  |
| Feb. 2025 | ND | 25% L925I, 50% L925I/I936F,  25% I936F | 320 ng/L^b^ | this study |
|  |  |  |  |  |

^a^LC_50_ for cyfluthrin

^b^LC_50_ for permethrin

^c^LC_50_ for bifenthrin
